# Ulcerative Colitis of the Neovagina in a Toddler with Cloaca and Chronic Kidney Disease

**DOI:** 10.1055/s-0041-1726868

**Published:** 2021-05-18

**Authors:** Marta Erculiani, Cinzia Zanatta, Enrico Vidal, Stefano Martelossi, Paola Midrio

**Affiliations:** 1 Division of Pediatric Surgery, Presidio Ospedaliero di Treviso, Treviso, Veneto, Italy; 2 Division of Pediatrics, Presidio Ospedaliero Universitario Santa Maria della Misericordia, Udine, Friuli-Venezia Giulia, Italy; 3 Division of Pediatrics, Presidio Ospedaliero di Treviso, Treviso, Veneto, Italy

**Keywords:** cloaca, neovagina, anorectal malformation, diagnosis, ulcerative, colitis

## Abstract

The case of a toddler with long-channel cloaca, mild chronic kidney disease (CKD) due to renal dysplasia, and early onset of ulcerative colitis (UC) is herein reported. The patient underwent definitive repair of cloaca, that included vaginal elongation with colon, at 5 months of age and was admitted for episodes of vaginal bleeding at 22 months of age. A vaginoscopy revealed a severe inflammation of the colonic neovagina. As rectal bleeding was also noticed, she underwent a colonscopy that showed the same macroscopic inflammatory picture. Neovaginal and colonic biopsies confirmed UC. The mother turned out to be affected by UC since adolescence. The patient is now on oral therapy with mesalazine and topical steroid and mesalazine in the neovagina. The association between cloaca and inflammatory bowel disease (IBD) is anecdotal, but the family history of IBD should be considered when planning the surgical reconstruction of patients with cloaca. In this patient, the occurrence of UC may require a new neovagina in the future and the concomitance of CKD may complicate the overall management due to the potential nephrotoxicity of drugs used for UC therapy.

## Introduction

Anorectal malformations (ARMs) comprise a wide spectrum of congenital anomalies that equally affect the genders and involve the distal anus and rectum as well as the urinary and genital tract. They occur approximately in 1 in 3,000 live births. Defects range from the minor and easily treated type with an excellent functional prognosis, to the complex and rare cases with poor functional prognosis. 1

The cloaca represents one of the rarest and most severe types of ARM in which the vagina, urethra, and rectum converge into a common channel and a single perineal orifice. 2 It is a very rare condition seen exclusively in females, with an estimated incidence of 1:50,000 births, 3 frequently associated with other anomalies, such as sacral, spinal, genitourinary, and cardiac malformations. To the best of our knowledge, the coexistence of cloaca and inflammatory bowel disease (IBD) affecting the colonic neovagina has been reported only once. 4

We report the case of a 22-month-old toddler with cloaca, mild chronic kidney disease (CKD) and onset of ulcerative colitis (UC) affecting the colonic neovagina at a very early age, thus a complicated course of the future multidisciplinary management is expected.

## Case Report

A female patient was born at 35 + 6 gestational weeks by C-section for abnormal cardiotocography and increasing hydrops. Bilateral hydronephrosis, bilateral hydrocolpos, ascites, and oligohydramnios were detected and monitored during pregnancy. At birth, the patient was hydropic, bradycardic, apneic, and cyanotic, requiring orotracheal intubation and mechanical ventilation. The birth weight was 4,510 g and Apgar scores at 1′/5′/10′ were 2/8/8, respectively. A laparotomy was immediately performed with drainage of both hydrocolpos through one vagina, as the septum was incomplete and the creation of a divided colostomy on the proximal sigmoid colon. A 12-French (Fr) feeding tube was left in one vagina to guarantee the constant drainage. At surgery, values of serum urea and creatinine were 7 mg/dL (normal values (n.v.) 5–18) and 0.85 mg/dL (n.v. 0.2–0.4), respectively.

During the next 2 weeks, renal function worsened, in association with bilateral hydroureteronephrosis, thus requiring a cystoscope-assisted insertion of an 8-Fr Foley catheter in the bladder. The renal function improved, and renal ultrasound showed mild bilateral hydronephrosis, with a slightly hyperechoic renal cortex. A contrast study through the Foley did not demonstrate the presence of a vesicoureteral reflux.

At 5 months of age, a cystovaginoscopy and subsequent posterior sagittal anorectal vaginal urethral plasty (PSARVUP) were performed. The cystovaginoscopy showed a 4-cm common channel, a 1.8-cm urethra, and the rectal fistula in the posterior wall of the left hemivagina. The PSARVUP included also a vaginal replacement with the sigmoid colon and the mobilization of the previous proximal colostomy to perform the anorectoplasty. Finally, a loop colostomy was opened on the transverse colon, the common channel tubularized to lengthen the urethra, and a suprapubic cystostomy was placed. The postoperative recovery was uneventful. Colostomy closure and cystostomy removal were performed at 7 months of age. Estimated glomerular filtration rate resulted in the lower limit of normal (86 mL/min/1.73 m ^2^ ).

**Fig. 1:**
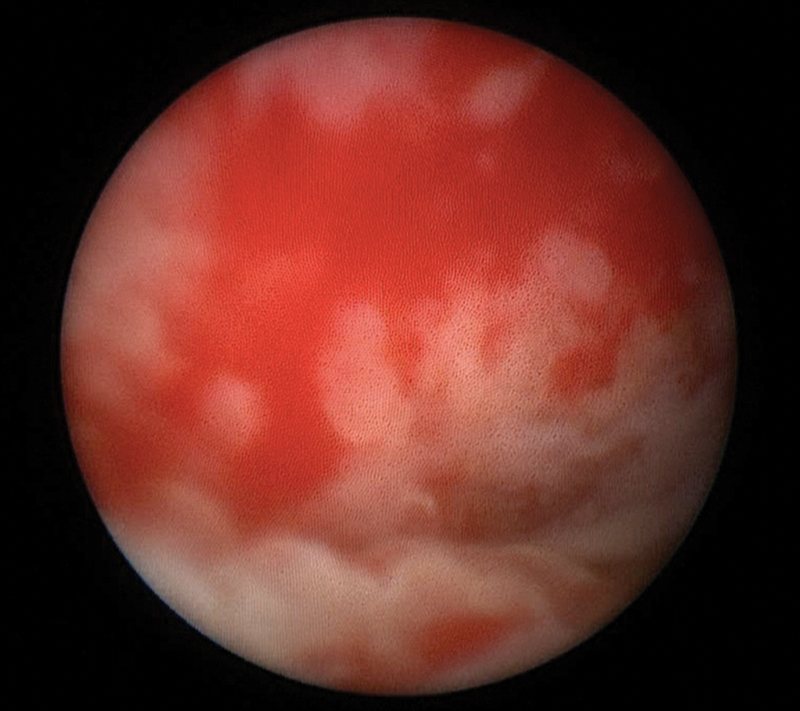
Neovagina's aspect.

**Fig. 2:**
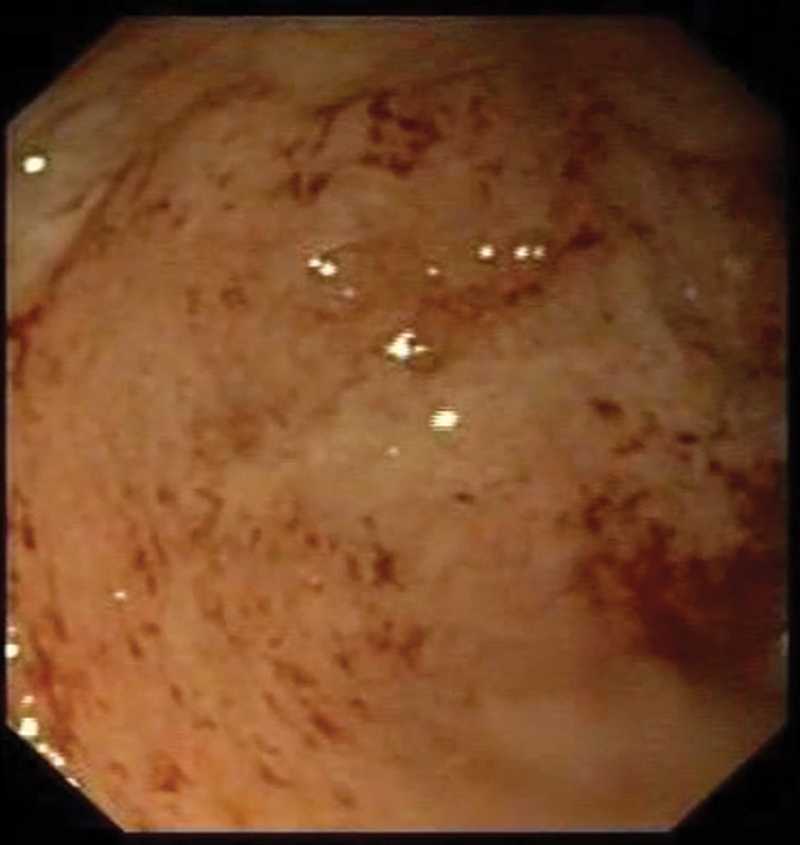
Colonic aspect.

At 22 months of age, she was admitted for repeated episodes of vaginal bleeding requiring blood transfusion. A vaginoscopy showed severe inflammation of the neovagina ( Fig. 1 ). As rectal bleeding was also noticed, a colonoscopy and retrograde ileoscopy were performed that showed the presence of inflammation up to the right colic flexure ( Fig. 2 ). Biopsies from both organs were suggestive for UC. Leukocytosis, elevated platelets count, low albumin, normal C-reactive protein, increased erythrocyte sedimentation rate, and fecal calprotectin 306 µg/g (n.v. 0–150) were detected. Pediatric ulcerative colitis activity index (PUCAI) was 15. At this time, the mother reported she was diagnosed with UC at 13 years of age.

The child started an oral therapy with prednisone and mesalazine, while monitoring the renal function, and topical mesalazine in the neovagina. After an initial good clinical response, 2 months after diagnosis, the vaginal bleeding reoccurred. The pediatric gastroenterologist decided to increase the oral mesalazine and to start topical steroid in the neovagina that promptly stopped the bleeding. At the ambulatory follow-up appointment with gastroenterologist 3 months after diagnosis, PUCAI was 0 and blood tests within normal ranges.

At the time of this report, the patient is 27 months old, asymptomatic, taking oral mesalazine and topical mesalazine and steroids. Her spine and vertebras are normal and her voiding pattern, although not completely mature, seems normal.

## Discussion

IBD affecting a colonic neovagina is an extremely rare event, previously reported in only six adults and two children. 4 5 6 7 8 9 10 11 These latter two cases included a patient with cloaca who developed UC at 34 months of age 4 and another with cloacal exstrophy who developed Crohn's disease of the neovagina and augmented bladder at 7 years of age. 11 Patients with UC have a familiar history of IBD in 8 to 14% of cases and first-degree relatives have four times the risk of developing the disease. 12

Although the etiopathogenesis of UC is not fully understood, it is suggested to be a complex interaction between genetic and environmental factors, host immune response, and gut microbiota. 13 In particular, UC seems to result from a homeostatic imbalance between host's mucosal immunity and enteric microflora, resulting in an aberrant response to nonpathogenic enteric bacteria. 14 Among environmental factors, food is reported as a possible trigger of UC; mechanisms through which food seems to potentially induce intestinal inflammation include direct dietary antigens, changes in the gut microbiome, and effects on gastrointestinal permeability. 15 The present case, indeed, developed UC in the colon graft placed outside the gastrointestinal tract, therefore not exposed to food antigens and with a microflora likely different from the enteric one. This fact may be explained by the involvement of the neovagina as a consequence of a systemic autoimmune response of all colonic tissue to an exposure in the colon.

Most children with UC present with diarrhea, rectal bleeding, and without systemic signs. Moreover, in pediatric patients, the pancolitis at onset is quite common with a natural course often more severe than for their adult counterparts. 16 Kelley-Quon et al 17 reported as predictive factors for colectomy a serum albumin less than 3.5 g/dL, significant weight loss at the time of diagnosis, and a first-degree relative with UC. Our patient had two of these predictive factors for colectomy in the future, and this, considering the underlying complex genitourinary–rectal malformation, is of a great concern. Whether an ileoanal anastomosis in a patient with already compromised fecal continence is better than a permanent ileostomy remains an open question.

Moreover, refractory recurrence of neovagina's bleeding could force to perform a new vaginal replacement, together with colectomy, with further uncertain outcome. The main surgical procedures for vaginal reconstruction are the inlay skin-graft technique, the use of grafts (peritoneum, bladder mucosa, amnios), and small or large bowel interposition. The latter one is more complex, but when performed by a trained team, it ensures a very low morbidity and complications. 8 When an isolated intestinal segment is used for vaginal reconstruction, the sigmoid colon is usually preferred over ileum because of its caliber and spontaneous mucous production, mimicking the vaginal tissue. 9 The ileum can also be used, but its mucosa produces more abundant and less lubricating secretions than the sigmoid segment and it often bleeds during intercourse.

For selected cases of cloaca, Bischoff et al 18 described a technique alternative to vaginal replacement, which is, the vaginal switch maneuver (VSM). The VSM can be considered if two big hemivaginas are present and located very high in the pelvis. The maneuver consists in resecting one hemiuterus and switching the dome of that hemivagina down to the perineum, preserving the blood supply to the ovary. The vaginal septum is resected and both hemivaginas are then tubularized as a single unit. However, this technique is affected by a high incidence of complications and need for vaginal replacement and, therefore, has become less popular over the years. Family history of IBD should be accurately evaluated in the risk/benefit ratio when planning the surgical reconstruction in children with cloaca to choose the better technique for the vaginal replacement.

A large proportion of patients affected by cloaca also have associated congenital anomalies of kidney and urinary tract that may affect the bladder and renal function. The structural renal anomalies most commonly identified are solitary kidney (13–26%), renal dysplasia (13–27%), ectopic kidney (8–14%), duplex kidneys (4–9%), hydronephrosis (16–58%), and ureteropelvic junction obstruction (5–8%). 19 Renal involvement is one of the extraintestinal manifestation of IBD that affects 4 to 23% of adults and 1 to 2% of children 20 and can present also as nephrolithiasis, tubulointerstitial nephritis, glomerulonephritis, and amyloidosis. 21 Many medications used in the treatment of IBD are known to have nephrotoxic adverse effects. In particular, 5-aminosalicylates (5-ASAs), that are commonly used in UC, can be nephrotoxic. 22 In many cases, it remains unclear whether the renal impairment in IBD patients is primary or secondary to drug side effects. The present patient is affected by a mild CKD due to a bilateral kidney dysplasia and takes oral therapy with 5-ASA. These factors might further impact on kidney function; therefore, a multidisciplinary long-term follow-up, including the pediatric gastroenterologist and nephrologist, is mandatory. The involvement of a gynecologist is also mandatory in all cloacae, especially in this case that is likely to experience a complicated gynecological course.

Last but not least, based on our experience, the psychological support of parents and patients is extremely important to optimize any treatment strategy in the future.

## Conclusion

The association between cloaca and IBD is anecdotal, but the family history of IBD should always be carefully investigated when planning the surgical reconstruction of complex urogenital malformations. This case, indeed, confirms that IBD can develop in a colon graft placed outside the gastrointestinal tract. In our patient, the concomitance of CKD complicates the overall management due to the potential nephrotoxicity of drugs used for UC therapy. Moreover, in case of failure of medical management, the patient may require a new neovagina and, possibly the total colectomy, thus raising significant questions about her overall quality of life.

## Notes

New Insights and the Importance for the Pediatric Surgeon
**The association between cloaca and IBD is anecdotal, but the family history of IBD should always be carefully investigated when planning the surgical reconstruction of complex urogenital malformations.**
